# Chronic Intoxication from the Habitual Use of the Essences, as Wermuth, Absinthe, Etc.

**Published:** 1891-12

**Authors:** 


### CHRONIC INTOXICATION FROM THE HABITUAL USE OF THE ESSENCES, AS WERMUTH, ABSINTHE, ETC.

Professor Lancereaux, in his lecture, says that it may take some time before the evil effects show themselves, from six to eight months to several years, and it is marvelous that so many women fall into the bad habit of taking their bitters. Poisoning by Absinthe, Amer., Picon, etc., resembles chronic alcoholism, but differs again in many symptoms. Thus one of my patients, who drinks Absinthe to excess, complained for a long time of cramps at night and of mucosities in the morning. Nightmares plague him constantly. He complains of severe pains in the extremities, especially mornings, and cough for a year. Formerly robust he is now emaciated, lost appetite. He suffers from excessive hyperalgesia all over the lower extremities, and hypogastrium. Tickling the sole of the foot is so painful that the patient twinges and twists about in his bed, and the mere touch of the skin there provokes a vivid reaction. There are spots, corresponding to the ovarian points, which are also extremely painful. Nightmares and cramps at night, in the morning dizziness, so that he would fall if not supported, nausea and vomiting of a thick, glairy mucus, which relieves him. Pulmonary tuberculosis will gradually finish him.

In another ward we have a woman of thirty-five years, menstruated at sixteen, married at twenty-one to a drunken brute, who introduced her into gin-mills, so that she enjoys, several times a day, her Absinthe. She coughs, looks pale and downhearted, her lower extremities are livid and her toes sweaty. The least tickling of the feet causes great pain, so that she hides herself in the bed. This hyperalgesia extends with her also to the upper extremities, to the abdomen, thorax. Plantar reflex highly exaggerated. Stitches and itching in the feet with the sensation as if a thousand needles pierced her toes, which often become benumbed, so that she does not feel them any more. Sleep restless on account of frightful dreams, and wakes up unrefreshed; disgust for meat, and vomits her food when coughing, for she has already a cavity in the apex of her right lung.

This excessive exaltation of sensitiveness, especially of the lower extremities, is characteristic of Absinthe, and it is symmetrical and ascending to the trunk, and yet it is a curious fact that pressure of the abdominal wall causes not only excessive pain, but also twisting of the head, contraction of the facial muscles, and torsion of the trunk, as in hysteria. Pressing the skin of the thorax on either side of the sternum causes excessive pain in the spinal cord where the nerves emerge. Reflex excitability is greatly exaggerated; from the least painful impression the muscles rapidly contract. At a later period we meet the anæsthesia so common to alcoholists, also symmetric with diminution of reflex excitability, for tickling the soles fails to produce flexion of the legs; still, even in advanced cases, the pain on pressure of the abdomen, thorax, and vertebral column persists in the same intensity as formerly. Subjective complaints are tingling, burning, stitching, worse by the heat of the bed, so that the sufferer often cries out at night and prevents all sleep. The patient sighs for death as a relief. Women often suffer the most. Some complain of sensation of oppression and constriction about the sternum, as if a weight would crush their chest or of the hysterical ball. Sight is often affected; the patient sees sparks, *muscæ volitantes;* luminous objects, red or yellow, later black or opaque, swimming before eyes, so that reading is impossible. To the amblyopia mental troubles follow, and a symmetrical paralysis, beginning in the feet and progressing upward, so that it may even affect the respiratory centre. Hallucinations are plenty, mostly painful and terrifying, affecting even hearing, at first at night, but soon they hear also the same voices when awake in daytime. At a late period the mind is severely affected, memory fails, and all intellectual labor becomes impossible, they laugh or weep without cause, the sphincters fail, and death ends the scene.

During the first stages something might be done, and our object must be to procure sleep by Morphine and Chloral, as thus the sufferings diminish. This must be followed by hydro-therapeutics for several months, until health and strength gradually return, and the use of these poisonous mixtures strictly prohibited.—*Bulletin Médical,* 15, '91.

We can do no better than recommend to our readers Gallavardin's treatise on drunkenness, or the chapter on drunkards in the third edition of Lilienthal's *Therapeutics.* Even after stopping the evil do not neglect to examine the lungs, for phthisis pulmonalis is too often the accompaniment or the sequelæ of continued debauchery. S. L.

